# USING ACTIGRAPHY TO ASSESS CHRONOTYPE AND PHYSICAL ACTIVITY IN OLDER ADULTS

**DOI:** 10.1093/geroni/igac059.2390

**Published:** 2022-12-20

**Authors:** Hilary Hicks, Genna Losinski, Pilar Thangwaritorn, Alex Laffer, Amber Watts

**Affiliations:** University of Kansas, Lawrence, Kansas, United States; University of Kansas, Lawrence, Kansas, United States; University of Kansas, LAWRENCE, Kansas, United States; University of Kansas, Lawrencee, Kansas, United States; University of Kansas, Lawrence, Kansas, United States

## Abstract

Chronotype refers to the time of day that people prefer to be active or to sleep and varies predictably across the lifespan. In younger samples, the morning-chronotype is related to greater levels of physical activity (PA) and improved health outcomes. It is unclear whether this pattern holds in older adults, a group that commonly exhibits an “early bird” preference. We investigated differences in PA patterns between chronotypes in 109 older adults (Mage = 70.45 years) using wrist-worn ActiGraphs in a free-living environment. ActiGraphs captured data about PA and sleep using a novel approach to measuring chronotype with the mid-point of the sleep interval. We categorized participants as morning-, intermediate-, or evening-chronotypes. We used ANCOVA to predict total and average peak PA from chronotype, adjusting for age, sex, education, and BMI. Total PA significantly differed between chronotypes such that evening-types engaged in less PA than both morning- and intermediate-types, F (2,102) = 4.377, p =.015. Average peak activity did not differ between chronotypes, p =.112. Consistent with findings in younger samples, our evening type participants engaged in less overall activity. A unique finding was that evening-types did not differ from their morning- and intermediate-chronotype peers in peak activity levels. This implies a key distinction between total activity and peak activity levels consistent with recent trends in PA research using a 24-hour-a-day framework instead of average or total activity levels. Future research should consider whether these differences in activity patterns translate into meaningful differences in health benefits in this age group.

